# Neuroprotective properties of levosimendan in an in vitro model of traumatic brain injury

**DOI:** 10.1186/1471-2377-10-97

**Published:** 2010-10-21

**Authors:** Anna B Roehl, Marc Hein, Philipp D Loetscher, Jan Rossaint, Joachim Weis, Rolf Rossaint, Mark Coburn

**Affiliations:** 1 Department of Anaesthesiology, RWTH Aachen University Hospital, Pauwelsstraße 30, 52074 Aachen, Germany; 2 Institute of Neuropathology, University Hospital of the RWTH Aachen, Pauwelsstraße 30, 52074 Aachen, Germany

## Abstract

**Background:**

We investigated the neuroprotective properties of levosimendan, a novel inodilator, in an in vitro model of traumatic brain injury.

**Methods:**

Organotypic hippocampal brain slices from mouse pups were subjected to a focal mechanical trauma. Slices were treated after the injury with three different concentrations of levosimendan (0.001, 0.01 and 0.1 μM) and compared to vehicle-treated slices. After 72 hrs, the trauma was quantified using propidium iodide to mark the injured cells.

**Results:**

A significant dose-dependent reduction of both total and secondary tissue injury was observed in cells treated with either 0.01 or 0.1 μM levosimendan compared to vehicle-treated slices.

****Conclusion**:**

Levosimendan represents a promising new pharmacological tool for neuroprotection after brain injury and warrants further investigation in an in vivo model.

## Background

Traumatic brain injury (TBI) is common, carries high rates of morbidity and mortality and lacks specific treatment. In our study of TBI, the initial lesion results from direct mechanical damage at the impact site. Subsequently, several cellular and molecular processes expand the local injury. This so-called secondary injury is due to several factors: excitotoxicity; mitochondrial dysfunction resulting in the up-regulation of cell-death genes and the formation of free radicals; and proapoptotic mediator pathway activation [1]. At present, medical intervention cannot rescue directly traumatised, dying cells. Therefore, current neuroprotective drugs target the surviving cells near the impact site [2]. Hypotension, hypoxia, hyper- and hypocapnia, and hyper- and hypoglycemia remain potentially avoidable insults, all of which aggravate the outcomes of TBI [3]. Levosimendan is a novel inodilator that enhances myocardial performance without leading to substantial changes in oxygen consumption. Levosimendan's positive inotropic and vasodilator effects are tied to its abilities to increase calcium sensitivity and open ATP-sensitive K+ channels (mitoK_ATP_channels) [4]. In a swine model of cardiac arrest, levosimendan significantly improved the initial resuscitation success, increased coronary perfusion pressure and elevated regional brain oxygen saturation [5]. Levosimendan favourably affects mitochondrial adenosine triphosphate synthesis, conferring cardioprotection and possible neuronal protection during ischemic insults. In a model of spinal cord injury, levosimendan has been reported to attenuate neurologic motor dysfunction [6]. This finding is supported by the fact that the selective mitoK_ATP_channel opener, diazoxide, is an effective neuroprotectant, as has been demonstrated in an ischemia reperfusion study in rats [4]. In fact, it has been observed that the secondary injury following a traumatic brain injury is similar to the post-ischemic neuronal damage observed in the penumbra surrounding the ischemic core after a stroke [7]. In the present study, we tested the hypothesis that levosimendan would provide neuroprotection for these selectively vulnerable neurons in an in vitro, organotypic, hippocampal slice model of cerebral trauma.

## Methods

**Figure 1:**
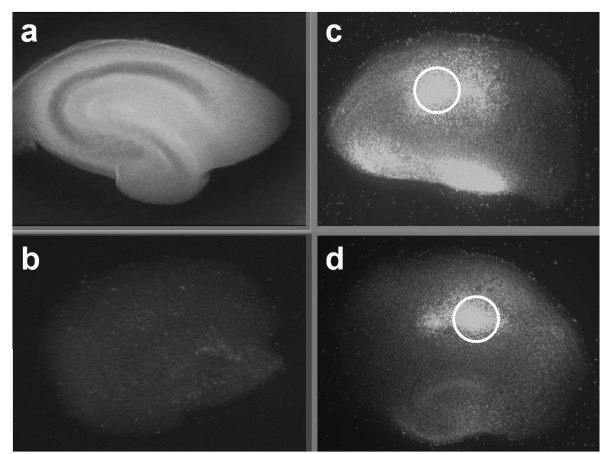
**After preparation, cultivation for 14 days and baseline measurement, slices were traumatised by dropping of a stylus onto the CA**_**1 **_**region of the hippocampus**. Figure 1a shows a native hippocampal slice prior to treatment. Figure 1b shows a non-traumatised, and un-treated negative control slice. The open circle tags the region of primary impact in Figure 1c and d. The treatment with 0.1 μM levosimendan (d) reduced visible the trauma intensity compared with the un-treated control (c).

All experiments were performed in compliance with the local institution's Ethical Review Committee and were approved by an animal protection representative at the Institute of Animal Research at the RWTH Aachen University Hospital, in accordance with German Animal Protection aw §4, Section 3. Organotypic hippocampal slices were prepared as reported previously, using the brains from six- to eight-day-old C57/BL6 mouse pups (Charles River Laboratories, Sulzfeld, Germany) [2,8,9]. Unless otherwise stated, all chemicals were obtained from PAA Laboratories GmbH (Pasching, Austria). The slices were maintained in culture for 14 days before experimentation. Traumatic brain injury was produced using a specially designed apparatus, which was also as previously reported [8]. Under stereomicroscopic supervision, a 1.65-mm diameter stylus was positioned 7 mm above the CA1 region of the hippocampal slices with the aid of a three-axis micromanipulator; the stylus was then dropped onto the slice with constant and reproducible impact energy of 5.26 μJ. For the experimental groups, the medium was exchanged immediately after trauma with an experimental medium containing levosimendan (Simdax^®^, 2.5 mg/ml, Orion Pharma, Espoo, Finland) at concentrations of 0.001 μM (n = 41), 0.01 μM (n = 41) and 0.1 μM (n = 52). Injured, untreated slices were considered the control group (n = 30). The slices were cultivated for 72 hrs in an incubator with an atmosphere of 95% air and 5% carbon dioxide at 37°C, after which cell death was quantified by measuring the intensity of propidium iodide (PI) fluorescence in the CA1 area (Figure 1).

**Figure 2:**
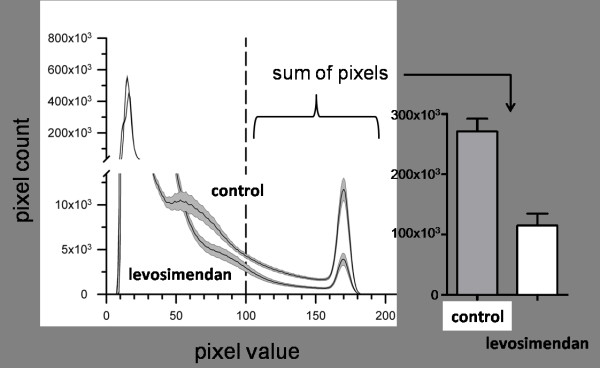
**Cell injury was quantified by measuring propidium iodide (PI) fluorescence as it entered only cells with damaged cell membranes**. The extent of the trauma was evaluated by fluorescence imaging 72 hours after trauma was induced, using pixel-based analysis of the resulting images. The control curve shows the histogram of un-treated slices (n = 30). The second curve shows the histogram of traumatised slices treated with 0.1 μM levosimendan (n = 52). The shaded region around each line indicates the SD. The extent of the injury was quantified by integrating the area at the sections where the pixel value fell above the threshold of 100.

Two types of tissue injury were examined: "total injury" was defined as the complete injury over the slice, whereas "secondary injury" described the injured area on the slice that excludes the primary impact site of the stylus. The relationship between the cumulative fluorescence emissions in PI-treated tissues and the number of damaged cells when compared to cell viability was assumed to be linear. Tissue injury in the slices was measured by pixel-based image analysis using ImageJ software (NIH; USA, http://rsb.info.nih.gov). This method has been proved effective in several previous studies [2,8-10]. The fluorescence images were digitalised at eight bits, allowing us to classify the images on a spectrum of 256 (from 0 to 255) greyscale levels. Damaged regions with high PI uptake emitted fluorescence at a high greyscale level, while vital regions showed only minor emissions. The red channel of each image was analysed with ImageJ software. For each image, ImageJ generated a histogram that showed the absolute number of pixels sharing the same greyscale value. Histograms from non-traumatised slices showed that the vast majority of all pixels had greyscale values between 10 and 100, representing mostly background fluorescence. In contrast, traumatised slices showed, in addition to their background fluorescence, a well-defined peak of values between 160 and 185 (Figure 2). As in previous publications [2,8,9], we established a threshold (in this instance, at a greyscale value of 100) that proved to be valid for distinguishing between traumatised and non-traumatised cells. Integrating the area under the histogram curve for all pixel values exceeding the threshold then allowed us to quantify cell injury.

To calculate the extent of the secondary injury, ImageJ was used to create a mask with the same diameter as the stylus. The mask was positioned directly over the stylus' impact site in the images, and that area was then excluded from the pixel analysis and subsequent calculations of trauma. The same mask was applied to every image when calculating the secondary injury. All values were normalised in reference to the control injury, which was defined as 100%. Both the mean value and the standard deviation (SD) were calculated for the trauma intensities of the slices in each group. One-way analysis of variance (ANOVA) with Bonferoni post-hoc analysis was used to test for statistical significance. A P-value of less than or equal to 0.05 was set as the threshold for statistical significance (SPSS 17.0, SPSS Inc., Chicago, IL, USA).

## Results and Discussion

**Figure 3:**
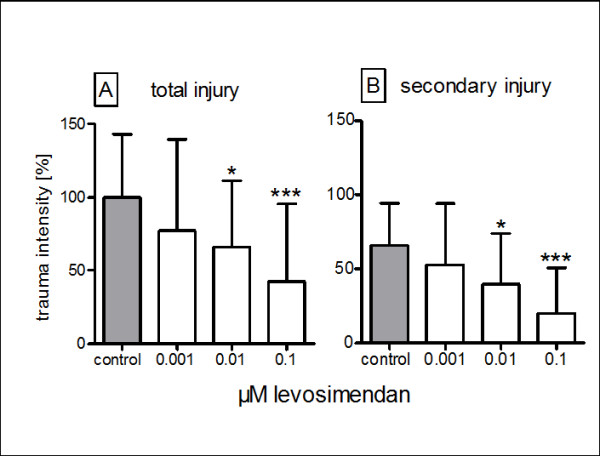
**The effect of levosimendan on the total (A) and secondary (B) posttraumatic injuries in mouse-derived organotypic hippocampal slices: each bar represents the mean ± SD normalised in reference to untreated slices (control)**. * p ≤ 0.05, p*** ≤0.001 vs. control from ANOVA and post-hoc analysis.

We found that levosimendan significantly diminished both total (p = 0.000) and secondary (p = 0.000) injury when administered in this in vitro model of traumatic brain injury. There appeared to be a dose-dependent neuroprotective effect in the observed range (between 0.001 and 0.1 μM) of levosimendan concentrations. Total injury in the experimental groups was significantly reduced relative to the control injury: to 66 ± 7% (p = 0.04) at 0.01 μM and to 42 ± 7% (p = 0.000) at 0.1 μM levosimendan (Figure 3A). The secondary injury, which accounted for approximately 66 ± 5% of the total injury, was reduced to 40 ± 4% (p = 0.009) and 20 ± 5% (p = 0.000) of the total control injury when treated with 0.01 μM and 0.1 μM levosimendan, respectively (Figure 3B). The reduction observed in the group treated with 0.001 μM levosimendan was not statistically significant for either total or secondary injury.

The precise mechanisms by which mitoK_ATP_channel activation protects the brain are unclear. One possible explanation is that the acute activation of mitoK_ATP _channels results in K^+ ^influx, organelle depolarisation, and the expansion of mitochondrial matrix volume. Mitochondrial Ca^2+ ^overload has been closely correlated with mitochondrial damage, which can result in both necrotic and apoptotic forms of cell death [4]. Thus, despite the profound differences in cellular physiology and sensitivity to anoxic injury that exist between myocardial cells and neurons, the same pharmacological approach has been shown to protect the heart [11] and, possibly, the brain, thereby saving both brain and heart tissues. The occurrence of focal injury at the primary site of impact and the subsequent development of secondary injury distant from the site are also comparable to the in vivo situation. Thus, this model can be confidently used as the testing environment for experimental treatments. In fact, we found that the secondary injury following traumatic brain injury displayed many similarities to the post-ischemic neuronal damage that can be observed in the penumbra surrounding the ischemic core following a stroke [7]. This likeness suggests that similar neuroprotective strategies may be successful in both etiologies of brain injury. The nature of the model excludes mechanisms of injury that are specific to brain damage in the in vivo situation, such as injury pathways related to brain swelling inside an enclosed skull, reperfusion injury, global or local ischemia, global or local hypoxia and other systemic variables [9]. Another limitation of this study is that levosimendan was administered directly following traumatisation, thus failing to account for the effects of delayed treatment that may be encountered in the routine clinical management of patients with traumatic brain injury, global ischemia or local ischemia.

## Conclusions

The present in vitro study demonstrated that levosimendan worked as an effective neuroprotectant in an in vitro model of traumatic brain injury. Levosimendan reduced both the total tissue injury and the secondary injury distant from the primary site of brain injury. The effects were observed at two different concentrations (0.01 and 0.1 μM) of levosimendan.

## Competing interests

The authors declare that they have no competing interests.

## Authors' contributions

AR and MH conceived of the study, participated in the study's design and coordination, performed the statistical analysis and drafted the manuscript. PL and JR conducted the experimental laboratory work. RR and JW helped to draft the manuscript. MC conceived of the study, participated in the study's design and coordination and helped to draft the manuscript. All authors read and approved the final manuscript.

## Pre-publication history

The pre-publication history for this paper can be accessed here:

http://www.biomedcentral.com/1471-2377/10/97/prepub
